# Mycotic Aortic Arch Aneurysm Caused by Clostridium perfringens

**DOI:** 10.7759/cureus.21135

**Published:** 2022-01-11

**Authors:** Atsumi Oishi, Tohru Asai, Kan Kajimoto, Yuki Kamikawa, Atsushi Amano

**Affiliations:** 1 Department of Cardiovascular Surgery, Juntendo University School of Medicine, Tokyo, JPN

**Keywords:** sepsis, mycotic aortic aneurysm, computed tomography, cholangitis, antibiotics

## Abstract

We report a case of a 79-year-old man for a mycotic aortic arch aneurysm caused by *Clostridium perfringens*. The patient who had been hospitalized for cholangitis two months prior revisited the hospital for fever and left precordial pain. He was suspected of an infected aortic aneurysm in the distal arch due to emphysematous changes observed. After antibiotics treatment, the emphysematous changes disappeared. However, he underwent urgent total arch replacement due to a new ulcer-like projection and enlargement of the aortic aneurysm, which were observed at that time. Clostridium-infected infectious aneurysms require not only treatment for vascular lesions but also scrutiny of complications, such as cancer.

## Introduction

Infected aortic aneurysm is a relatively rare disease that accounts for 0.5-1.3% of all aortic aneurysm cases [1]. However, the complications, which include sepsis, multiple organ failure, and rupture of the aortic aneurysm, give it a higher mortality rate compared with non-infected aortic aneurysms. Additionally, mycotic aortic aneurysm is extremely rare and accounts for 0.5-2% of all infected aortic aneurysm cases [2,3]. Often intractable, it is a deep-seated mycosis that requires comprehensive treatment including surgery, antibiotic medication, and treatment for the complications associated with systemic sepsis.

Mycotic aortic arch aneurysm caused by *Clostridium perfringens *is a life-threatening disorder. In fact, the mortality rate is 100% if untreated [4]. Herein, we report a patient with a mycotic aortic arch aneurysm caused by *C. perfringens* who subsequently underwent a semi-urgent total arch replacement.

## Case presentation

This study was approved by the institutional review board at Juntendo Hospital, Juntendo University School of Medicine (approval number: JHS 21-039; approval date: November 4, 2021), and the patient provided written informed consent.

A 79-year-old man who had been hospitalized for cholangitis two months prior revisited the hospital for fever and left precordial pain. Physical examinations revealed a fever of 39.1°C, blood pressure of 184/90 mmHg, heart rate of 128 beats/minute, respiratory rate of 24 breaths/minute, and oxygen saturation of 90% on room air. White blood cell (WBC) count was 20100/μL and C-reactive protein was 11.70 mg/dL. The patient was diagnosed with cholangitis and was started on 6 g/day of ampicillin/sulbactam (ABPC/SBT).

**Figure 1:**
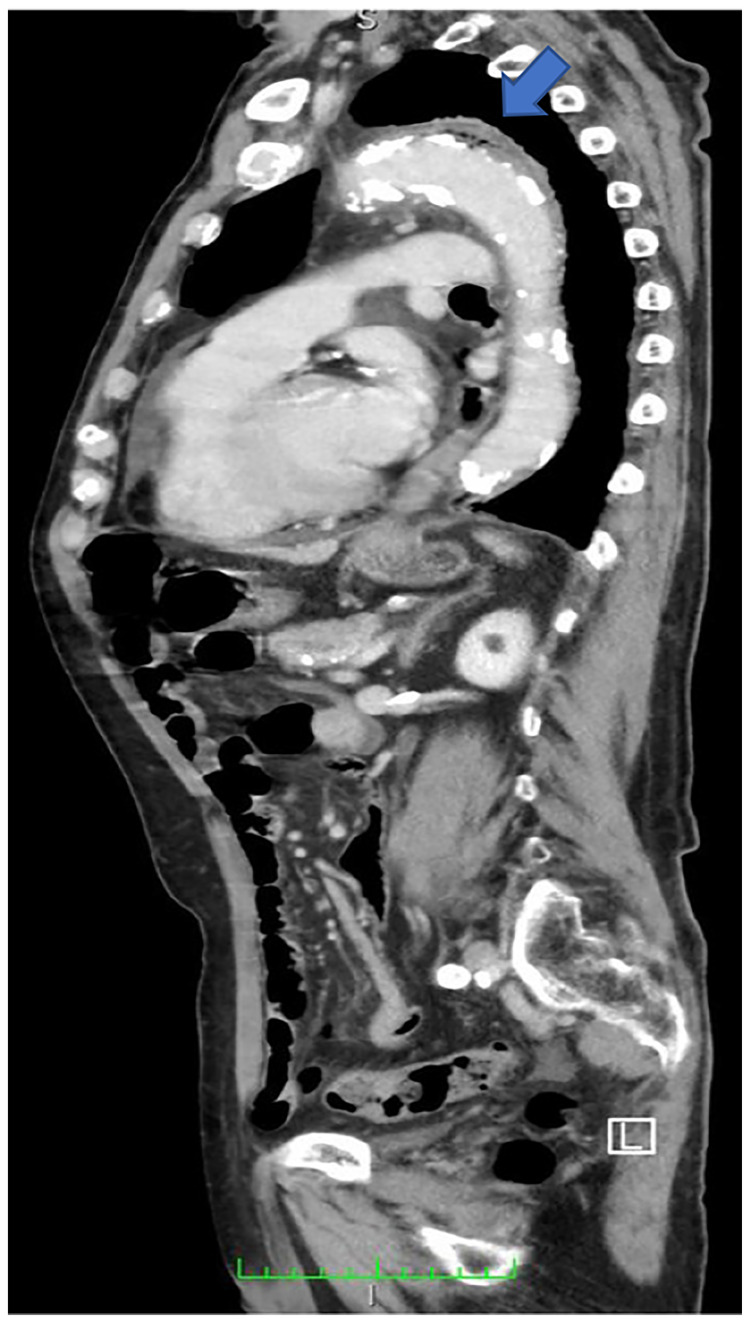
Chest computed tomography (sagittal view) showing emphysematous aortitis.

Aortic aneurysm was not observed on chest x-ray. However, enhanced computed tomography (CT) showed emphysematous changes in the distal aortic arch (Figure 1). Additionally, blood cultures were positive for *C. perfringens*. Thus, treating team changed the extended-spectrum antibiotics from ABPC/SBT to 3 g/day of meropenem (MEPM). The patient was further evaluated by echocardiography. The finding of echocardiography showed normal left ventricular function and no vegetation.

**Figure 2:**
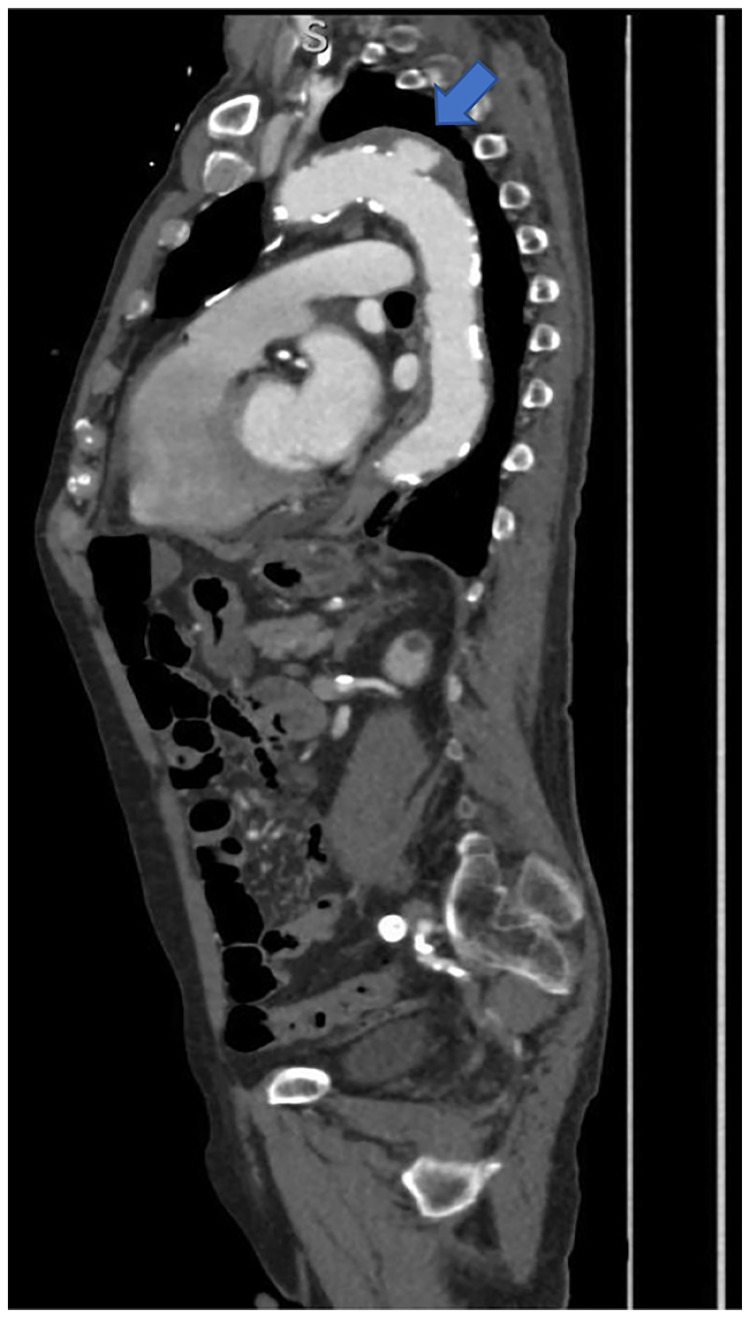
Chest computed tomography (sagittal view) showing ulcer-like projection.

Another enhanced CT conducted after 13 days of antibiotic treatment showed the disappearance of the emphysematous changes and ulcer-like projections (ULP) were noted (Figure 2). The patient was transported to our hospital with a diagnosis of clostridial mycotic distal aortic arch aneurysm for surgical treatment. Vital signs on admission were as follows: body temperature was 36.7°C, blood pressure was 105/64 mmHg, heart rate was 69 beats/minute, respiratory rate was 18 breaths/minute, and oxygen saturation was 94% on nasal cannula 2 L.

**Figure 3:**
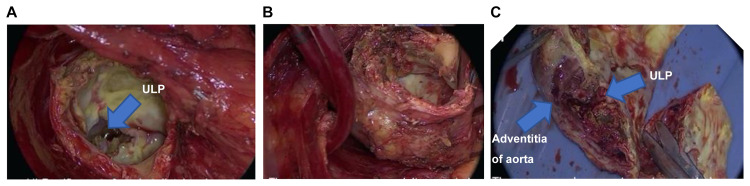
Intraoperative photograph of aneurysm. (A) Orifice of the ulcer-like projection found at distal aortic arch. (B) Removal of the aneurysm. No abscesses were noted. (C) The aneurysmal segment was transected. Necrosis, thrombus, and edema were prominent.

The surgery was conducted on a semi-urgent basis. After establishing cardiopulmonary bypass, hypothermia (below 25°C tympanic membrane temperature) induced circulatory arrest and selective cerebral perfusion were achieved. The distal aortic arch at the infected site was removed to the level of the left pulmonary artery, including adhesions. Infected tissue were completely resected. The anastomoses were carried out carefully to prevent injury of fragile aortic wall and avoided using artificial materials including felt strip. Total arch replacement was performed with a Gelweave graft (Shibuya, Tokyo: Terumo Corporation) which had soaked for 15 minutes in a solution of rifampicin (1 mg/mL of saline), and the left atrial appendage was excised. Aortic regurgitation was mild on trans-esophageal echocardiography, but strong calcification was observed in the left coronary cusp, which was thus removed. The ULP in the distal arch had a red thrombus that created a dissociation cavity from the intimal defect, and acute inflammation at about 1 mm from the adventitia, along with edema of the tissue, were observed, to which the surrounding lung was completely attached (Figures 3A-3C).

The operation was completed in about four hours, as scheduled. Good hemodynamics and emergence from anesthesia were confirmed on the day of the operation without any problems in brain and myocardial protection. The patient was extubated in the morning of the first postoperative day (POD) and was back on his feet by the next day.

On POD seven, removal of common bile duct stones was performed by endoscopic retrograde cholangiopancreatography. On POD 10, inflammatory response improved and we switched back the medication from MEPM to ABPC/SBT. On POD 15, the patient was transferred to the previous hospital to continue antibacterial treatment and rehabilitation.

## Discussion

The symptoms of the acute phase of *C. perfringens* infection are reported as severe chest pain, abdominal pain, lower back pain, fever, and other symptoms. In the present case, the patient experienced severe chest pain due to cholangitis or diverticulitis caused by the infection. Atherosclerotic plaque and vascular calcification or intimal damage due to smoking were possible risk factors for infected aneurysms in this patient.

As *C. perfringens* infection is associated with complications of colorectal or hematologic malignant tumors, blood tests, CT, or endoscopy are essential to confirm or rule out the diagnosis in the case of suspected infection [5]. Whole-body CT should be performed for patients with clostridial infections to check for aortic inflammation or infection. Characteristic features of Clostridium-infected aortic aneurysms are emphysematous changes within and around the aorta as well as the rapid expansion of aneurysms and ULP. Mortality from clostridial mycotic aortic aneurysms can be reduced by early diagnosis and comprehensive treatment, which include surgical intervention before the rupture and early administration of appropriate antibiotics.

Endovascular treatment of aortic aneurysm is a less invasive approach compared with thoracotomy or laparotomy [6]. Despite reports of successful endovascular treatment of infectious aortic aneurysms, doubts remain on the efficacy of the technique to treat infected lesions. However, it may be a good option for high-risk patients who are unsuitable for thoracotomy and laparotomy or for ruptured aneurysms and life-threatening situations.

Rapid antibiotic administration is another critical point in the treatment of clostridial infections. The first-choice antibiotic is penicillin, and the second-choice antibiotics are third- and fourth-generation cephalosporins, metronidazole, imipenem, and vancomycin. These drugs should be used in combination with other drugs so that they can be applied widely. Prolonged antibiotics treatment should also be administered for at least six to eight weeks after surgery. Without intervention, the prognosis is poor and the mortality rate is 50-100% [7].

The limitation of this study is the undetermined duration of antibiotic administration after discharge. Intervention for infected aortic aneurysms is more likely to save lives by combining prompt diagnosis and early appropriate antibiotic administration with timely surgical treatment.

## Conclusions

Treatment of Clostridium-infected aortic aneurysms is extremely difficult. In order to improve the outcome of patients diagnosed with this rare infection, it is considered that early diagnosis by identification of ULP or emphysematous changes, appropriate early antibiotic treatment, and rapid surgery may reduce mortality.

Clostridium-infected infectious aneurysms require not only treatment for vascular lesions but also scrutiny of complications, such as cancer. When there are complications, it is a disease that requires management and comprehensive treatment, not only by surgery but also by a cross-sectional team.
